# Unemployment and Substance Use: An Updated Review of Studies from North America and Europe

**DOI:** 10.3390/healthcare11081182

**Published:** 2023-04-20

**Authors:** Carina Nolte-Troha, Patrik Roser, Dieter Henkel, Norbert Scherbaum, Gabriele Koller, Andreas G. Franke

**Affiliations:** 1 Department of Psychiatry and Psychotherapy, University Hospital Munich, Ludwig-Maximilians-University Munich, Nußbaumstr. 7, 80336 Munich, Germany; 2 Department of Psychiatry and Psychotherapy, LVR University Hospital Essen, University of Duisburg-Essen, Virchowstr. 174, 45147 Essen, Germany; 3 Main Institute of Addiction Research (ISFF), University of Applied Sciences Frankfurt, Nibelungenplatz 1, 60318 Frankfurt am Main, Germany; 4 University of Applied Labour Studies, Seckenheimer Landstr. 16, 68163 Mannheim, Germany

**Keywords:** unemployment, substance-use disorders, addiction, drugs

## Abstract

Since the industrial revolution, the relationship between unemployment and psychiatric disorders has been a subject of high interest. Currently, regarding the correlation between unemployment and substance-use disorders (SUDs), only older, often isolated and fragmented research results are available in the literature. This review was based on an extensive literature search of the European and North American literature in most relevant databases for “unemployment” and “substance use” related to “drugs”, “alcohol”, “nicotine”, and “tobacco” between November 2022 and January 2023, according to the PRISMA (Preferred Reporting Items for Systematic review and Meta-Analysis) guidelines. A total of 59,117 papers were identified, of which only 33 articles were identified as relevant to the research objective. The literature showed significantly higher prevalence rates of SUDs involving divergent psychotropic substances among unemployed people. Unemployment was found to be a risk factor for SUD, and vice versa. However, the correlation between unemployment and relapses or smoking cessation was inconsistent. In addition, there appeared to be a mild effect of business cycles on SUD. The results showed significant multifaceted correlations between unemployment and SUD, indicating that prevention and early intervention are required to prevent harmful psychosocial consequences, such as social disintegration and severe psychiatric disorders.

## 1. Introduction

The relationship between unemployment and health problems has been investigated for decades. The first study focusing on unemployment and health was the Marienthal Study, which investigated a small Austrian village surrounding a textile factory that had been shut down due to the global economic crises in the 1930s [1]. For the first time, the Marienthal Study demonstrated that mass unemployment was associated with health problems and additional psychosocially problematic aspects. This has been confirmed by a plethora of studies and reviews, as well as meta-analyses in Germany [2,3,4,5], while others have provided broader international contexts [6,7,8,9,10].

Later studies have often discussed the causal direction: Is unemployment leading to mental disorders or are mental disorders leading to unemployment [11,12,13,14]? However, both causal directions have appeared to be possible [11,13]: The first suggested that losing one’s job and becoming unemployed led to loss of income and, thus, psychosocial stress, all of which worsen the parameters of good health and may lead to (short-term) coping strategies, including substance use. The second causal direction indicated that having mental disorders led to increased sick leave, reduced productivity, and decreased overall psychosocial, educational, and (eventually) employment opportunities, leading to an increased likelihood of being or becoming unemployed.

Current research results have revealed and divided somatic as well as mental health consequences of unemployment. However, a specific focus on substance-use disorders (SUDs) is still significantly underrepresented in the research, and at times, it has even been excluded from systematic reviews [15]. Despite this lack of data, SUD is one of the most frequent psychiatric disorders and is an important public health concern worldwide [16,17,18,19].

Initially, the research regarding unemployment and SUD was focused on alcohol use. Eventually, other psychotropic substances, such as tobacco as well as illicit and prescription drugs, were studied within the context of unemployment. The above-mentioned causal direction has been an important aspect in this field as well, and researchers have focused on the relationship between SUD and the business cycle, as well as the unemployment rate [20,21].

The majority of studies in this field have focused on divergent but isolated aspects, such as depression, schizophrenia, substance abuse, addiction, etc. Of the current research, literature reviews and meta-analyses make up only a fraction of the published work (e.g., [14,22,23]). Therefore, it is difficult to gain an accurate overview of the current research results concerning mental disorders in general and their association with unemployment.

Therefore, in 2011, Dieter Henkel published an extensive literature review summarizing studies on unemployment and substance use between 1990 and 2010 to answer six specific questions:

1. To what extent are substance use and SUD affecting the unemployed versus the employed?
2. To what extent does substance use and SUD increase the likelihood of unemployment and decrease the chances of employment or being rehired?
3. To what extent is unemployment a risk factor for SUD?
4. Does unemployment increase the risk of relapse after treatment?
5. Does unemployment reduce the success of smoking cessation?
6. To what extent are substance-use patterns associated with unemployment rates and are there cyclical fluctuations?

Dieter Henkel’s summary of the literature, up until 2010, was the basis of the literature review presented here. The aim of the present review was to provide an update regarding the relationship between unemployment and substance use and SUD, with an emphasis on the situation of North America and Europe.

## 2. Materials and Methods

To achieve the goal of this study, an extensive review of the literature was performed. The entire procedure was performed according to the PRISMA (Preferred Reporting Items for Systematic review and Meta-Analysis) guidelines [24], as follows:

This study was conducted by two of the authors (CNT and AGF) between October 2022 and January 2023, and they performed an extensive search of the literature using the following databases: Elsevier, Emerald Insight, Pub Med Central, Research Gate, Springer Link, Thieme Connect, and Wiley Online Library. These databases were accessed online using Ludwig Maximilian University (LMU) Munich’s online public access catalogue (OPAC), which provided access to all the aforementioned databases. Of the choices offered in OPAC (i.e., “OPAC university bibliotheca catalogue” and “OPAC scientific articles and more”), we chose the “OPAC scientific articles and more” for our research.

The inclusion criteria for the studies were 1. matching the search terms, and 2. publications written in English. The following search terms were used: “unemployment” and “substance use” related to “drugs”, “alcohol”, “nicotine”, and “tobacco”. For filtering and adjusting, quotation marks were used.

In summary, four search terms were used independently from the others: “unemployment substance use drugs”, “unemployment substance use tobacco”, “unemployment substance use nicotine”, and “unemployment substance use alcohol”. Subsequently, only publications written in English were chosen, which were marked by a cross in the “OPAC scientific articles and more” section.

**Figure 1:**
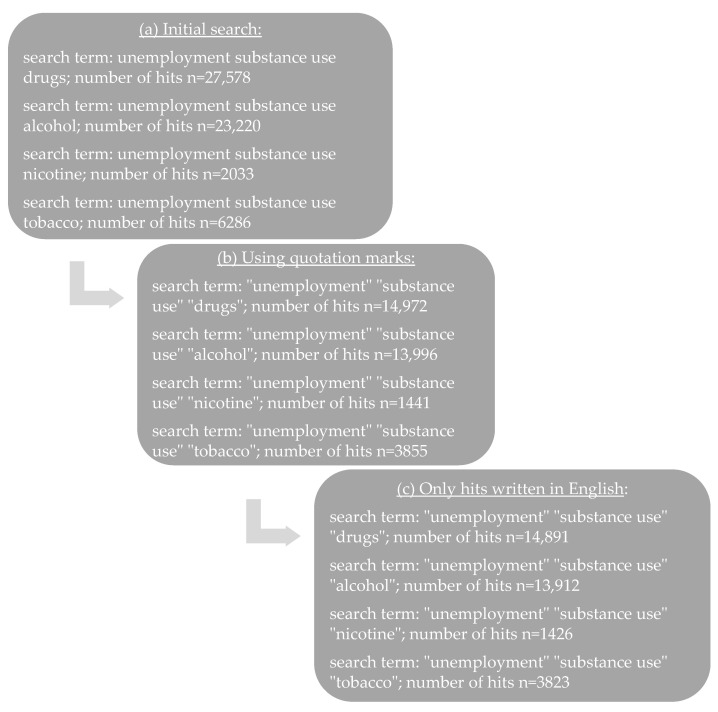
Search strategy and results of literature search between August 2010 and January 2023. (**a**) Initial search terms used, (**b**) quotations marks added, and (**c**) only texts written in English.

All search terms were applied to the years between 2010 and 2023. Furthermore, filtering was performed to ensure that only meaningful articles had been screened. The procedure is illustrated in Figure 1. Since Henkel’s review ended in August 2010, only studies published after that date were included in our research.

Additional articles and population-based surveys were searched via Google and Google Scholar, using the same search terms (“unemployment substance use drugs”, “unemployment substance use tobacco”, “unemployment substance use nicotine”, “unemployment substance use alcohol”) independently. The years of publication could not be filtered by the initial search; however, publication prior to August 2010 was excluded in a second step.

In a second step, all abstracts were screened by the same reviewers (CNT and AGF), and publications that did not meet the inclusion criteria (e.g., unemployment, substance use) were excluded from further consideration.

Once the literature search had been completed, the results were compared and discussed. The publications listed in the next section were identified as being relevant to this article by both reviewers.

## 3. Results

Based on the literature published between August 2010 and January 2023, 59,117 articles were found, as shown in Figure 1. After this initial search, all studies were excluded that did not meet the inclusion criteria. The results were screened by two reviewers who excluded all studies that met the inclusion criteria but were not relevant to the subject of this review. A total of 33 papers were identified as applicable to the objective of this review.

### 3.1. Prevalence of SUD among the Employed and Unemployed

Over the past 12 years, several studies have been published concerning the epidemiological and sociodemographic aspects of SUD use and employment that had significant implications for prevention and treatment needs. These studies included diverse sample sizes, ranging from 87 participants to 2.7 million [25,26] from different countries in the U.S. and Europe (e.g., France, Denmark, Germany, etc., [27,28,29]) with diverse characteristics (age [30], gender [31], education [32], race [33], etc.), and with different types of unemployment scenarios (short-term, long-term, etc., [34]) and various employment histories (numerous jobs, inconsistent employment, continuous unemployment, etc.). Researchers have used different statistical analyses (e.g., descriptive statistics [29], logistic regression analysis [34], etc.), different diagnostic criteria and manuals (Diagnostic and Statistical Manual of Mental Disorders (DSM), International Classification of Disorders (ICD); Comprehensive International Diagnostic Interview [35], Diagnostic Interview Schedule [36], Structured Clinical Interview (SCID) for DSM-IV [25,37,38], etc.), and different definitions of substance-use patterns (e.g., binge drinking, heavy drinking, hazardous drinking [27,39]).

Furthermore, the vast majority of studies focused on alcohol, followed by tobacco and cannabis (see Table 1); only a small proportion studied other drugs, such as cocaine and opioids [25,40].

The main results of the literature search are presented in Table 1.

Regarding SUD, most studies revealed significantly higher rates of substance abuse, SUD, and addiction for unemployed people, as compared to employed people (see Table 1).

Regarding alcohol use, unemployed people showed a significantly higher frequency in their use of alcohol than employed people. In addition, they consumed slightly higher amounts of alcohol. In addition, unemployed people had significantly higher rates of problematic or “high-dose” drinking patterns. Furthermore, unemployed males had higher frequencies of drinking alcohol than employed women. One study, confirming the survey results, was based on somatic markers, such as carbohydrate-deficient transferrin, aspartate transaminase, etc. [41]. Another study from Finland used alcohol-related deaths as markers, which were more frequently found among the unemployed, as compared to employed people [42].

In addition, unemployed people had significantly higher prevalence in smoking and vaping. Moreover, tobacco use by smoking was the most frequently reported behavior (regarding the use of psychotropic substances) of unemployed people, as compared to employed. The unemployed smoked more frequently and in greater quantities (number of cigarettes), as well. The frequency and amount of tobacco use appeared to be stable during unemployment [30]. Smoking was more frequent in males than in females, which was comparable to employed people.

Regarding illegal drugs such as cannabis, the results were similar: Unemployed people used cannabis more frequently than employed people. A single Spanish study found opposite results; however, there were no data about the amount of cannabis used. However, the results for cocaine were not as clear as for the other drugs: A study from the U.S. revealed similar prevalence rates in both groups, with a higher number of previous therapeutic interventions for cocaine use among the employed, as compared to unemployed people [43]. Regarding methamphetamine use, unemployment was associated with a more frequent use of methamphetamines and a longer duration of using methamphetamines [44].

Regarding gender differences among unemployed people, males had higher prevalence rates for SUD than females. The same (higher prevalence rates among males, as compared to females) was valid for employed males and females. However, for sedative use, females showed higher prevalence rates than males among employed as well as unemployed people [32].

Regarding covariates, the statistical analyses of some studies showed that the following aspects were important covariates: age, gender, educational level, occupational status, income, marital status, race, ethnicity, and rural vs. urban residence [31]. Throughout the studies, unemployed people had a lower socioeconomic status than employed people.

Apart from the use of (hypno-) sedatives, there were no data on the prevalence of the use and misuse of prescription drugs.

**Table 1:** Prevalence of Substance Use Disorders Among Unemployed (U) and Employed (E), Prevalence Rates (%), Odds Ratios (OR).

Data Source	Authors	N, Age	Substance-Use Disorders	U (%)		E (%)		OR E = 1
U.K. MCS/ ALSPAC/ NS/BCS70/ NCDS/ USOC/ ELSA/GS 2020	Green, M. et al., 2022 [45]	27,841 16–66	Current smoking (furloughed, no longer employed, stable unemployed) Current vaping (furloughed, no longer employed, stable unemployed) Current drinking (furloughed, no longer employed, stable unemployed)					
France CONSTANCES 2012/2018	El Haddad et al., 2022 [46]	1427 18–69	Tobacco use					
			Light smoker	19.4		14.2		
			Moderate smoker	6.5		7.3		
			Heavy smoker	2.2		0.6		
			Cannabis use					
			>12 months ago	13.0		13.0		
			<1x per month	8.1		8.1		
			≥1x per month	16.2		8.1		
			Alcohol use					
			Frequency [0–2/3–5/6+ AUDIT sub-score]	34.6/33.0/32.4		32.2/38.2/29.6		
			Dependence [1,2/3+ AUDIT sub-score]	29.7/24.3		30.6/19.6		
Denmark DNHS 2010	Egan, K. et al., 2021 [47]	84,474 18–60	Median alcohol consumption (5th–95th)	7.13 drinks/week		9.1 drinks/week		
			Problem drinking	5.7		2.8		
			Current smoker	35.5		18.1		
Netherlands DLCS 2006–2013	Mangot-Sala, L. et al., 2021 [34]	45,967 25–50		Short U	Long U			
			Abstainer	16.71 ***	22.86 ***	15.15 ***		
			Moderate drinking (<1.5 drinks/day)	71.75	62.60	71.59		
			Heavy drinking (≥1.5 drinks/day)	11.54	14.55	13.26		
			Binge drinking (≥5 [♂]/≥4 [♀] drinks/occasion)	12.50	13.07 *	10.86 *		
USA TEDS 1993–2016	Azagba, S. et al., 2021 [48]	n.a. ≥18	Treatment admissions for primary substance abuse (opiates, cocaine, alcohol, marijuana, other drugs, stimulants)					
Sweden SWIP 2010–2017	Jonsson, J. et al., 2021 [26]	2,743,764 18–61	Substance abuse	♂97/♀8 cases; Adjusted HR *****: 1.0		♂6902/♀5245 cases in standard employment; Adjusted HR: 2.5		
England GP 2011–2017	Vandoros, S. et al., 2020 [49]	8736 n.a.	Opioid prescribing					
			Defined daily dose per capita	1.745				
			Items per capita	0.102				
			Quantity per capita	9.526				
			Defined daily dose per 1000 days	19.108				
USA CARDIA 2005–2010	Swift, S. et al., 2020 [50]	1307 n.a.	Binge drinking, past 30-day drug use					
Italy n.a. 2019	De Sio, S. et al., 2020 [41]	314 n.a.	Carbohydrate-deficient transferrin	13.4		7.0		0.47
			Aspartate transaminase	23.6 **		12.1 **		0.42 **
			Alanine transaminase	55.7 **		34.8 **		0.39 **
			γ-glutamyltransferase	29.3 *		17.8 *		0.48 *
			Mean corpuscular volume	23.6 **		10.8 **		0.37 **
Spain EDADES 2007/2013	Casal, B. et al., 2020 [40]	23,258/ 22,862; 16–64		2007	2013	2007	2013	
			Cannabis use	13.52	34.23	56.40	36.63	
			Cocaine use	15.87	37.45	62.19	44.57	
			Both	16.49	39.61	59.72	40.82	
Denmark DNHS 2010	Bæksgaard Jørgensen, M. et al., 2019 [51]	86,417 18–60	Median alcohol consumption (5th–95th)	10.0 drinks/week		7.6 drinks/week		
			Problem drinking	5.1		2.8		
			Current smoker	38.1		23.0		
France CONSTANCES 2012–2016	Airagnes, G. et al., 2019 [52]	18,879 18–69	Alcohol use					
			Dangerous	16.4		11.5		1.46 ***
			Problematic or dependence	3.4		1.5		1.92 ***
			Tobacco use					
			Light smoker	14.3		10.5		1.54 ***
			Moderate smoker	10.0		6.6		1.69 ***
			Heavy smoker	3.5		2.1		1.78 **
			Cannabis use					
			>12 months ago	43.9		39.5		1.45 ***
			<1x per month	6.5		4.0		1.87 ***
			≥1x per month	8.8		4.0		1.68 ***
USA n.a. n.a.	Miguel, A. et al., 2019 [43]	553 ≥18	History of cocaine use					
			Age of cocaine onset	20.6 ± 5.6 years		21.3 ± 7 years		
			Years of cocaine use	10.3 ± 7.9 years		8.8 ± 7.7 years		
			Days of cocaine use in 28 days prior to treatment	14.5 ± 8.6 days		14 ± 9.1 days		
			Pretreatment cocaine positive urine result	72.8		77.2		1.61
			History of substance abuse treatment					
			Treatment naïve	16.4		15.6		
			Number outpatient substance-use treatments	2.2 ± 3.3 treatments		2.4 ± 3.4		
			Number inpatient substance-use treatments	2.7 ± 4.3 treatments		2.9 ± 5.9		
			Concomitant psychiatric disorders					
			Lifetime alcohol-use disorder	68		68.3		
Germany SUNRISE 2021	Scherbaum, N. et al., 2018 [25]	87 25–49	Alcohol abuse/addiction	5.8/69.8				
			Opioid abuse/addiction	2.3/4.7				
			Opioid dependence, but in substitution treatment	10.5				
			Cannabis abuse/addiction	0.5/26.7				
			Sedative/hypnotics abuse/addiction	4.7/7.0				
			Cocaine abuse/addiction	7.0/4.7				
			Stimulants abuse/addiction	7.0/2.3				
			Hallucinogen abuse	2.3				
			Polydrugmania	1.2				
Spain EDADES 2013	Teixidó-Compañó, E. et al., 2018 [32]	14,113 25–64	Hazardous drinking	♂7.9; ♀2.6		♂6.2; ♀3.9		
			Heavy cannabis use	♂6.2; ♀1.5		♂2.4; ♀0.6		
			Hypnosedative consumption	♂9.9; ♀18.4		♂6.4; ♀13.4		
USA NESARC 2001–2004/ 2012–2013	Okechukwu, C. et al., 2018 [38]	23,446/ 23,346 ≥18		2012–13		2012–13		
			Past year marijuana use (daily/weekly/ ≤monthly)	22.95 (7.56/4.62/10.77/77.05)		10.27 (3.02/1.89/5.36)		
			Past year use disorder	6.65		2.63		
			Lifetime alcohol disorder	47.71		33.04		
			Lifetime drug disorder	12.20		5.79		
Netherlands HIS 2004–2013	De Goeij, M. et al., 2017 [53]	26,355 30–64	Episodic drinking					
			Before economic crisis (2004–2008)	1.3				0.87
			During economic crisis (2008–2013)	2.9				1.24
USA PSID 1999–2011	Grafova, I. et al., 2017 [31]	41,231 n.a.	Current smoker	♂19.87; ♀18.52				
			Former smoker	♂30.69; ♀27.55				
			Smoking relapse	♂8.09; ♀10.20				
			Increased cigarette consumption	♂14.24; ♀15.98				
			Quit smoking	♂17.19; ♀17.93				
			Decreased cigarette consumption	♂42.50; ♀43.04				
Spain Flash EB 2011/2014	Ayllón, S. et al., 2018 [29]	n.a. 15–24		2011	2014			
			Consumption of cannabis					
			At any point in time	24.0	28.5			
			Last 30 days	5.0	5.7			
			Last 12 months	7.4	8.7			
			>12 months ago	11.6	14.1			
			Consumption of new substances					
			At any point in time	4.7	7.4			
			Last 30 days		1.1			
			Last 12 months		2.2			
			>12 months ago		4.0			
Germany SUF 2016	Hollederer, A. et al., 2016 [27]	8951 15–64	Consumption of beer, wine or mixed drinks					
			every day	♂9.1; ♀0.3		♂7.9; ♀1.4 ***		
			several times a week	♂14.2; ♀4.2		♂24.1; ♀10.6 ***		
			once a week	♂16.0; ♀9.7		♂26.9; ♀15.3 ***		
			less common	♂32.7; ♀36.0		♂31.0; ♀50.6 ***		
			Consumption of spirits					
			every day	♂1.2; ♀ <0.1		♂0.2; 0.0 f ***		
			several times a week	♂1.9; ♀0.2		♂2.5; 0.7 f ***		
			once a week	♂6.6; ♀1.7		♂12.7; 2.4 f ***		
			less common	♂36.3; ♀20.5		♂45.7; 35.1 f ***		
			Nicotine consumption	♂66.0 ***; ♀52.1 ***		♂32.2 ***; 25.6 f ***		
AT/BE/CZ/DK/FR/DE/IT/NL/ES/SE/CH ****** SHARE 2006–2012	Bosque-Prous, M. et al., 2015 [39]	7615 50–64	Hazardous drinking (≥40 g and ≥20 g of pure alcohol per day)	7.3		7.1		
USA SSDP n.a.	Lee, J. et al., 2015 [54]	n.a. 22–33	Heavy episodic drinking (>6 months of use) Daily cigarette smoking (>6 months of use) Marijuana use (>6 months of use)					
USA FTDO 1992–1994	Chintakrindi, S. et al., 2015 [55]	n.a. n.a.	Drug use prior to arrest (used alcohol, marijuana, uppers, cocaine, crack, heroin, any drugs)					
Finland SF 2000–2007	Paljärvi, T. et al., 2014 [42]	204,422 45–64	Alcohol-related death in relation to average annual number of days	177.5 days		134.9 days		
USA NSDUH 2002–2004/ 2005–2007/ 2008/ 2009–2010	Compton, W. et al., 2014 [28]	~ 405,000 ≥18		2009–10		2009–10		
			Heavy alcohol use (≥5 drinks on ≥5 days)	0.58		0.16 ****		1.17
			Illicit drug use	0.66		0.18 ****		1.60
			Tobacco use	0.98		0.31 ****		1.56
			Alcohol abuse/dependence	0.57		0.16 ****		1.28
			Illicit drug abuse/dependence	0.37		0.08 ****		1.79
USA n.a. 1983/ 1985–1986/ 1992/1997/ 2002/2007/ 2012–2013	Brook, J. et al., 2014 [56]	2012–13 528 ∅43						2012–13
			Heavy/continuous smokers					3.84 *
			Late starters					1.57
			Occasional smokers					4.03 *
			Quitters/decreasers					1.51
USA NAS12 2009–2010	Mulia, N. et al., 2014 [33]	5382 ≥18	Alcohol-related health problems prior to recession	14.6				
USA NESARC 2001–2002/ 2004–2005	Baldwin, M. et al., 2013 [57]	n.a. 19–60				Part-time	Full-time	
			Alcohol disorder (not employed, employed part-time, and employed full-time in each wave 1 and 2)	39.67		34.26	86.63	
			Drug disorder (not employed, employed part-time, and employed full-time in each wave 1 and 2)	45.88		21.26	83.34	
USA CLS 2004–2005	Arria, A. et al., 2013 [58]	620 17–19				Part-time	Full-time	
			Alcohol consumption	4.8		3.9	4.8	
			Drug use pattern group					
			Infrequent marijuana use	29.4		19.7	25.7	
			Sporadic drug use	35.5		35.5	41.0	
			Persistent drug use	29.4		21.1	14.3	
USA HRS 1992–2008	Deb, P. et al., 2012 [59]	20,557 ≥50	Daily number of drinks	0.818 drinks/day		0.811 drinks/day		
Germany SOEP 1998–2009	Schunck, R. et al., 2012 [30]	17,028 17–65	Smoking Number of cigarettes/day	48.94 17.08 cigarettes/day		33.34 16.60 cigarettes/day		
USA NESARC 2001–2002/ 2004–2005	Dávalos, M. et al., 2012 [60]	34,120 ≥18	Alcohol consumption (past year)					
			Any binge drinking	21.8/24.9				
			Days of binge drinking (≥5 [♂]/ ≥ 4 [♀] drinks per episode)	12.8 days/12.7 days				
			Driving after too much	2.7/3.7				
			to drink (≥1 in past year)					
			Abuse and/or dependence	7.7/9.0				
USA n.a. n.a.	Weber, E. et al., 2012 [44]	110 n.a.	Metamphetamine (MA) use characteristics					
			Age of first use	22.5 years		21.9 years		
			Total duration of use	11.4 years		7.7 years		
			Total quantity of use	4834 g		3704 g		
			Last use	139 days		188 days		
			Injection MA use ever	52.1 *		13.3 *		
			Non-MA dependence					
			Cannabis (lifetime)	10.4		20.0		
			Alcohol (lifetime)	35.4		40.0		
			Cocaine (lifetime)	20.8		33.3		
			Other substances (lifetime)	14.6		6.7		

MCS: Millennium Cohort Study, ALSPAC: Avon Longitudinal Study of Parents and Children (G0 = parents, G1 = children), NS: Next Steps, BCS70: 1970 British Cohort Study, NCDS: National Child Development Study, USOC: Understanding Society, ELSA: English Longitudinal Study of Aging, GS: Generation Scotland, AUDIT: Alcohol-Use Disorders Identification Test, DNHS: Danish National Health Survey, DLCS: Dutch Lifelines Cohort Study, TEDS: Treatment Episode Data Set, SWIP: Swedish Work, Illness, and Labour-market Participation, GP: General Practice Data Dashboard, CARDIA: Coronary Artery Risk Development in Young Adults, EDADES: Spanish Household Survey on Alcohol and Drugs in Spain, SUNRISE: Integrated Support of Unemployed at Risk of Substance Abuse Disorders, NESARC: National Epidemiologic Survey on Alcohol and Related Conditions, HIS: Health Interview Survey, PSID: Panel Study of Income Dynamics, Flash EB 401: Flash Eurobarometer 401 ‘Young people and drugs’, SUF: Scientific Use File, SHARE: Survey of Health, Ageing and Retirement in Europe, SSDP: Seattle Social Development Project, FTDO: First Time Drug Offender Program, SF: Statistics Finland, NSDUH: U.S. National Survey on Drug Use and Health, NAS12: U.S. National Alcohol Survey, CLS: College Life Study, HRS: Health and Retirement Survey, SOEP: German Socio-Economic Panel, * *p* < 0.05, ** *p* < 0.01, *** *p* < 0.001, **** *p* < 0.0001, ***** HR: Hazard Ratio ****** AT/BE/CZ/DK/FR/DE/IT/NL/ES/SE/CH: Austria, Belgium, Czech Rep., Denmark, France, Germany, Italy, Netherlands, Spain, Sweden, Switzerland.

### 3.2. SUD as a Risk Factor for Unemployment

It was demonstrated that, from a governmental perspective, SUD led to politico-economic losses and reduced economic productivity [61,62]. This was shown at a macro-scale. In this respect, several studies addressed the use of psychotropic substances and their effects on the labor market. However, these results were found within the contexts of economics and government policy.

The findings presented in Table 2 were more focused on addiction medicine, and they emphasized the objectives of this review: On average, we found that the current literature clearly indicated that problematic substance use increased the risk of unemployment. Furthermore, this also decreased opportunities for finding employment or being rehired [51]. In particular, this likelihood was shown in relation to alcohol use. Moreover, these effects were dose dependent: People who drank more alcohol, and those with problematic drinking habits, were more likely to become unemployed. In a study from Denmark, this correlation was not only valid for becoming unemployed but also for the decreased likelihood of those who were already unemployed returning to work [51]. Additional studies have shown that people with problematic substance use were more likely to lose their job and be unable to find employment later; this was supported by the results of a Danish study. A German study found that, on average, unemployment occurred 11 years after the first signs of SUD (independent from the type of substance) [25]. A French study showed these aspects for short-term unemployment [52]. A study from the U.S. showed that young adults suffering from alcohol-use disorder and additional infrequent illegal drug use consistently over time could have a higher risk of unemployment after college [58]. Another study investigated mixed substance use, and the authors found that alcohol, tobacco, and cannabis use all increased the likelihood of unemployment [52]. Results from Germany highlighted these findings, in addition to diagnoses of abuse and addiction of alcohol, opioids, cannabis, sedatives, hypnotics, cocaine, stimulants, hallucinogens, and polydrug use [25]. According to a French study, in particular, the use of cannabis and cocaine had significant effects on short-term unemployment [52]. In this respect, a study from the U.S. depicted that recent marijuana use (i.e., within the last year) increased the risk of job loss by 50% [38].

However, regarding legal drugs, a French study revealed that tobacco and risky alcohol use did not significantly affect employment (as compared to the weekly use of cannabis and alcohol dependence) [46]. In contrast, a study from the U.S. showed that continuous and occasional smokers tended to become unemployed in later years [56].

Regarding illegal drugs, methamphetamine dependence that led to neurocognitive deficits was associated with higher rates of unemployment [44].

The correlation between SUD and unemployment was valid over the life span of participants who were aged from 18 to 69 years.

However, the studies over the past 12 years did not address SUD and educational levels or other further aspects, which we describe in the Section 4.

### 3.3. Unemployment as a Risk Factor for the Development of SUD

As compared to other aspects of this review, studies on alcohol use were the most frequent studies that considered the development of SUD due to unemployment. However, there were some remarkable studies about tobacco use, cannabis use, and the use of other drugs. However, no studies on prescription drugs were identified.

Starting with alcohol, a study from the U.S. clearly revealed that job loss was associated with the higher risk of alcohol-related health problems (in males) (e.g., drunkenness) and the development of alcohol addiction [33]. This correlation was confirmed by most studies (see Table 2).

In addition, unemployment was associated with higher rates of tobacco and illicit drug use as well as alcohol and illicit drug dependence (see Table 3). A study from the U.S. confirmed this effect in young adults and their use of alcohol (heavy episodic drinking) [54].

However, a study from the Netherlands that included more than 45,000 participants did not show a correlation between job loss and alcohol use or SUD but revealed a more general correlation between being consistently unemployed over a long period and heavy as well as binge drinking [34]. Moreover, some short-term unemployed individuals were more likely to abstain from alcohol [34], which contradicted previous findings. A study from the U.S. showed that negative employment chances were associated with decreasing binge drinking but increasing drug use during the previous 30-day period [50]. A Spanish study found no correlation, however, and another study from the U.S. found that unemployment initially decreased the risk of cigarette use and relapse (in men) [31]. In this respect, the study only found a correlation between stable unemployment and females as well as for stable unemployment and smoking.

Unemployment among young adults was associated with heavy episodic drinking. This did not apply to marijuana use and was only marginally linked to daily cigarette smoking [54].

Regarding illegal drugs, stable unemployment during the treatment of cocaine use was associated with negative outcomes, while becoming employed during treatment was associated with better outcomes [43].

Regarding gender, a Spanish study showed that there was no effect on cannabis use and the abuse of sedatives, whereas unemployed females showed lower rates of alcohol use [32].

In summary, there was a trend for unemployment as a risk factor for developing SUD and even addiction. However, this trend was less clear than for question two (SUD being a risk factor for unemployment).

### 3.4. Unemployment as a Risk Factor for Relapses after Smoking Cessation

Regarding this question, there was a paucity of studies. The three available studies (see Table 4) addressed tobacco use. Regarding the first review by Green and their colleagues, five out of the seven surveys showed that unemployed participants had higher rates of smoking and vaping relapses [45].

The second study revealed that 10% of the unemployed participants who smoked in the past, prior to the first wave, relapsed until a second wave [31]. The third study showed that unemployed people did not appear to show changes in smoking relapse [30] and also had inconsistent results for tobacco use.

However, all three studies did not include data about the method of smoking cessation or data about the period of abstinence.

Unfortunately, studies about relapses regarding alcohol or other psychotropic drugs were not identified.

### 3.5. Unemployment Reducing the Success of Smoking Cessation

Although there was strong evidence regarding the high prevalence rates of smoking among unemployed people (see Table 1), there was little information available regarding the unemployed and smoking cessation (see Table 5).

Study results illustrated an ambiguous picture: On the one hand, studies showed a reduced probability of smoking cessation among unemployed people, but on the other hand, the results were contradictory. For example, a German study showed that unemployment was associated with a higher likelihood of smoking, but there were no differences among commencing, relapsing, or quitting [30]. An American study concluded that there was no significant difference between those who had stopped smoking and non-smokers in terms of unemployment [56]. The review of Green and their colleagues found that two-in-seven surveys showed that unemployed participants had lower rates of smoking and cessation, while one-in-six surveys indicated less vaping, including vaping cessation [45].

Unfortunately, no systematic trends were observed in the various studies included in this review.

### 3.6. Substance-Use Patterns Based on Unemployment Rates and Business Cycle/Cyclical Fluctuations

Unemployment increased the likelihood of SUD, and SUD increased the likelihood of unemployment. Therefore, the influencing factors had to be considered.

The macro-economic perspective, as well as the perspective of the labor market determinants, suggested that the business cycles or cyclical fluctuations could have an effect on substance-use patterns (see Table 6).

There were significantly fewer studies addressing this aspect, as compared to studies addressing the core field of addiction medicine, including prevalence rates, reasons for unemployment, and reasons for SUD (as previously described).

A Spanish study from 2020 showed that cannabis and cocaine use had a higher impact on unemployment, especially in economic recessions [40]. This study was aligned with another study from the Netherlands, which reported a retrospective analysis on the economic crisis of 2008, revealing that job loss during the economic crisis had been associated with chronic alcohol use but not with episodic drinking [53]. Another study demonstrated that the correlation between unemployment rates and annual substance abuse admissions had the same causal direction during economic crises and during times without crisis [48].

However, “systematic realist literature review” by Nagelhout and their colleagues showed that “most of the studies that did examine these latter mechanisms confirmed the hypothesized counter-cyclical associations” [63].

Despite the lack of evidence, in summary, it could be assumed that there was an increase in the use of psychotropic substances when the economy deteriorated and the unemployment rate increased, representing a counter-cyclical pattern.

## 4. Discussion

The present literature review provided an update regarding the context of SUD and unemployment. This update was established based on the literature published in the last 12 years concerning Europe and the U.S. It showed a correlation between unemployment and SUD. To address the six aforementioned questions in the Introduction, our review found the following: (1) Among unemployed people, there were higher prevalence rates for substance use and SUD for nearly all psychotropic substances, especially alcohol. This was valid independently for gender, age, and other independent factors. Furthermore, in general, the review found that (2) substance use and SUD were risk factors for unemployment and (3) vice versa: Unemployment was a risk factor for substance use. Moreover, (4) unemployment appeared to be a risk factor of relapses after treatment, and (5) unemployment appeared to reduce the success of smoking cessation. In addition, (6) a correlation between substance-use patterns and unemployment rates based on business cycle/cyclical fluctuations was shown according to the trends.

However, though our results roughly confirmed our hypotheses, some of the results varied significantly. This was not surprising and should be discussed further. An important superordinate aspect was that the studies reviewed had significantly different parameters for their characterizations, which could have resulted in significant biases. There were different sample sizes (less than 100, more than 2.7 million [25,26]) and sample characteristics (age, gender, social strata, etc. [30,31,32,33]) that led to the diversity in their results. Furthermore, the different diagnostic criteria and manuals used (e.g., DSM, ICD, different standardized diagnostic instrument, etc. [35,36,37]) could also have led to the underestimation of prevalence rates, as shown in the European Study of the Epidemiology of Mental Disorders (ESEMeD) of 2004 [64], which found relatively low prevalence rates of alcohol abuse and dependence. This could have been due to the use of more conservative definitions of alcohol disorders provided by the DSM-IV and an updated version of the Composite International Diagnostic Interview (CIDI).

Regarding the term “unemployment”, the studies used different definitions (e.g., short-term unemployment, unspecified unemployment, part-time employment, temporary employment), while other studies did not define this term at all. Only a few studies differentiated between full-time, part-time, and temporary employment [65,66,67]. For example, an older study showed that long-term unemployment was associated with a higher prevalence for SUD than short-term unemployment, which could have been related to the duration of unemployment [68].

Additional aspects that could have led to a wide range of results were different definitions of drinking and/or smoking patterns (e.g., [34,38]) and the studies being based on divergent drugs with different drug characteristics, consequences of use, etc. [69]. Moreover, substance-use patterns and the terms used to describe these patterns were not consistent, and the definitions of the terms “substance use” and “addiction” included a broad range of substance use characteristics that could be related to the wide range of their results [35,36,37,70].

In addition, different methods were used to ensure different degrees of anonymity for the participants and the data (different survey methods, interview methods, etc. [34]). Assuming that both unemployment and SUD were associated with social stigmas [71], this could have decreased the prevalence rates of SUD based on self-report [72]. Therefore, a higher estimated number of unknown cases had to be assumed, which could have limited the accuracy of the respective studies. This bias should be considered when addressing stigmatized issues. However, one study was based on somatic markers (e.g., carbohydrate-deficient transferrin, aspartate transaminase, etc.) and was, therefore, independent from stigmatization and socially desirable answers. This study confirmed other survey results [41].

In addition to stigmatization, the legal regulations of drug possession and drug use could have led to international, and even regional, differences [73,74,75], which could also have explained the wide range of the results.

Concerning question two (substance use and SUD as risk factors for unemployment), the studies demonstrated that SUD had a substantial negative effect on labor market outcomes, in general. The results based on the analyzed studies were consistent and compelling, as demonstrated in the Section 3. Furthermore, they were aligned with previous reviews about this topic [13]. However, the underlying mechanisms were not outlined in the studies we reviewed. The aforementioned effect could be due to a reduction in physical and mental health; reduced work performance and productivity; and increased absence from work due to illness, which were actually caused by the use of psychotropic substances (intoxication, rush, injuries, etc.), as shown in previous studies [61,76,77]. In addition, regarding illegal drugs, criminal activities could lead to psychosocial conflicts, legal conflicts with the police, and even imprisonment [73]. These aspects could explain the underlying mechanisms that could not be shown by the studies included in this review.

The third question investigated whether unemployment could lead to substance use. This question was affirmed, as well, in previous studies [13,78]. However, as before, the underlying mechanisms could not be explained. Therefore, two different hypotheses were under consideration: 1. Unemployment increased SUD, beginning with the shock of losing one’s job. This was followed by unemployment that was associated with negative psychosocial aspects, such as financial problems, identity crisis, monotony, etc., for which drugs were used as compensation [13,79]. This theory was aligned with the results of the present review. 2. Unemployment could lead to a decrease in SUD due to decreases in income (reduced availability of money) and work-related stress [80,81].

In addition, two types of job loss had to be considered: involuntary job loss, such as in the case of dismissal or redundancy, and voluntary job loss, in order to adopt a new profession or take advantage of a private opportunity. This assumption was supported by Ettner and their colleagues [82]. They found that the negative effects (higher prevalence rates of SUD) were only observed among those who had been dismissed and should be regarded as involuntary unemployment. The group with voluntary unemployment showed a significant decline of alcohol use [82]. Furthermore, the present study results could have been influenced by a phenomenon described by previous studies, describing that even the fear of job loss could increase SUD [83,84].

An older study by Deb and their colleagues provided evidence that individuals already using substances problematically prior to job loss were more likely to respond to job loss by increasing their substance use, and these further increases could be especially problematic [59].

In addition, future research should examine the role of income loss. To date, one study addressed this topic: The authors demonstrated that a loss of income did not influence the likelihood of becoming alcohol dependent (using DSM-III) [85]. However, in view of the significant income loss experienced by the unemployed, it was reasonable to assume that unemployed people would reduce their alcohol and tobacco use and, potentially, their use of other psychotropic substances.

Regarding the fourth question (unemployment as a risk factor for relapses after treatment), the literature search did not find studies concerning alcohol or illegal drugs in this aspect, but we did identify three studies focused on smoking relapse. The identified studies showed inconsistent results, however. As compared to previous findings, only one older study could directly demonstrate that unemployed people had a higher likelihood of relapsing and suffered from more severe relapses than employed people/patients [86]. Regarding alcohol, it was demonstrated several times that a significant proportion of in-patients were unemployed [87]. However, to answer this question, a longitudinal study design was necessary. At least two reviews and one meta-analysis confirmed that unemployment was a risk factor of relapses after treatment for a few psychotropic substances (alcohol, opioids) [88,89]. Further studies confirmed these results and found that those patients who remained unemployed after treatment were 2–3 times more likely to relapse than those who were employed [86]. Therefore, there was strong evidence that unemployment substantially increased the risk of relapse. Dieter Henkel concluded that a patient’s employment status was increasingly considered a crucial predictor of treatment outcome [13].

The fifth question (unemployment could reduce the success of smoking cessation) could only be answered according to the trends: Study results showed that smoking cessation was less likely among unemployed [30,56]. However, a review including seven studies only found two studies confirming the aforementioned finding [45].

In this respect, pharmacotherapeutic, behavioral, and psychosocial interventions have been effective for smoking cessation [90,91,92]. However, previous studies showing the effectiveness of treatment for smoking did not assess employment status. In addition, previous studies addressing with the question of smoking cessation and employment status did not show a consistent picture [93,94,95]. This was comparable to the results of our study. This revealed the need for more research in this field.

The last question (substance-use patterns based on unemployment rates and business cycle/cyclical fluctuations) was answered by the studies in this review, as the studies found a significant association between business cycles and employment rates and SUD as an anticyclic effect (upswing associated with low substance use, downturn associated with high SUD). This anticyclic effect was anticipated due to the higher unemployment rates reported during economic downturns, and high unemployment rates have been associated with higher prevalence rates of SUD (as shown above). This revealed an indirect effect and was aligned with previous studies that had shown anticyclical results [20,21]. However, there were studies with contradictory results, and some even showed a procyclic correlation [96,97,98,99]. Furthermore, there were studies that failed to find a procyclic or an anticyclic effect and produced inconsistent results [100,101].

These inconsistent results indicated that more research is needed regarding this correlation.

Beyond the aforementioned aspects, there were aspects that have not been addressed in the literature. For example, using prescription drugs prescribed by a physician or the misuse of such prescribed drugs are aspects that have not been addressed systematically. There were very few (two) studies that addressed prescription drug use [25,32]. Only a Spanish study showed results demonstrating higher prevalence rates for hypnosedative use by the unemployed [32]; the second study from Germany did not investigate the differences between employed and unemployed but focused on only the unemployed [25]. Previous studies (prior to 2010) and studies that did not meet the inclusion criteria demonstrated that unemployed people had higher prevalence rates of prescription drugs. This could have been explained by the fact that unemployed people have higher prevalence rates of mental disorders, which typically require psychoactive prescription drugs, such as (anxiolytic) sedatives and hypno-sedatives [102,103,104] for treatment. However, the studies were not systematically able to differentiate between the indicated use and the misuse of these prescription drugs.

The previously discussed aspects were of high importance on the subject of unemployment and SUD and their correlation. However, there was a gap in the literature concerning the systematic study of unemployment and SUD in the context of social and cultural patterns, norms, and lifestyle factors and habits. Henkel’s review did not present such data, either. Further studies should consider these likely important aspects.

In addition to the above, there were some methodological limitations: We had to assume that some data were not found due to a search strategy with (a) specific keywords that did not include all scientific studies, (b) specific databases that excluded others, and (c) a criterion that restricted studies to only those written in the English language. This may have led to a certain bias regarding the included studies. Specifically, using the keywords “alcohol”, “nicotine”, and “tobacco” favored studies containing these psychotropic substances, which, in turn, may have biased our results. However, as compared to illegal drugs, alcohol and tobacco have significantly higher prevalence rates than illegal drugs.

Furthermore, country-specific aspects, such as unemployment rates as well as national economic cycles, could not be considered systematically due to complications regarding economic evaluations and the longevity of business cycles, which could not all be considered in a single study. In this respect, the field of social and unemployment politics was not considered. Furthermore, countries differ in the aforementioned aspects, which may have influenced the study results.

In addition, the review provided an overview of Europe and North America while excluding other continents and countries. The results from Europe and North America could be heavily influenced by the highly industrialized and developed characteristics of these regions. Therefore, the results may not be representative or comparable to less-industrialized, developing countries and regions of the world.

## 5. Conclusions

The present review was inspired by the findings of Dieter Henkel in 2011 and provides an update on his findings in Europe and North America. It largely confirmed his results. This review demonstrated that there were significant correlations between unemployment and SUD, with significantly higher rates of SUD among unemployed people. Regarding relapses and smoking cessation among the unemployed, the results have been inconsistent. However, as compared to previous studies, we found that economic developments and cycles have some effect.

In this study, our correlations were multifaceted and complex. Therefore, our results should be interpreted carefully. In addition, some questions remain unanswered, especially those related to moderating and mediating variables and underlying mechanisms. More research is needed.

Therapeutic options, in general, are required for SUD, and based on our research, these options need to be customized to an individual’s characteristics and their specific situation in order to prevent addiction. Unemployment as well as SUD, and even addiction, are important factors leading to social difficulties for those affected, and they impose significant expenses at a national level. Looking at the proportion of people who struggle with unemployment and addiction in a society, those two factors may lead to societal strains and, potentially, even destabilize existing societal and cultural infrastructure.

## Figures and Tables

**Table 2:** Effects of Substance-Use Disorders on Unemployment/Employment.

Data Source, N, Age	Authors	Substance-Use Variables	Labor Market Outcomes	Effects
France, CONSTANCES 2012/2018, 1427, 18–69	El Haddad et al., 2022 [46]	Tobacco and cannabis use, alcohol frequency of use and dependence	Unemployment/ Employment	Tobacco and risky alcohol use do not significantly affect employment unlike weekly use of cannabis (OR 1.73) and alcohol dependence (OR 1.65).
Denmark, DNHS, 2010, 84,474, 18–60	Egan, K. et al., 2021 [47]	Median alcohol consumption, problem drinking, current smoker	Unemployment/ Employment	People who drink more alcohol up to problem drinking are more likely affected to become unemployed.
Spain, EDADES, 2007/2013, 22,862, 15–64	Casal, B. et al., 2020 [40]	Consumption of cannabis, cocaine and both of them	Unemployment/ Employment	Cannabis and cocaine use has an higher impact on unemployment, especially in economic recession.
Denmark, DNHS, 2010, 86,417, 18–60	Bæksgaard Jørgensen, M. et al., 2019 [51]	Median alcohol consumption, problem drinking, current smoker	Unemployment/ Employment	People who drink more alcohol up to problem drinking are more likely affected to become unemployed; moreover, they have a lower chance to return to work.
France, CONSTANCES, 2012–2016, 18,879, 18–69	Airagnes, G. et al., 2019 [52]	Consumption of alcohol, tobacco and cannabis	Unemployment/ Employment	All the substance-use variables show correlation with short-term unemployment.
Germany, SUNRISE, 2021, 87, 24–54	Scherbaum, N. et al., 2018 [25]	Abuse/addiction of alcohol, opioids, cannabis, sedatives, hypnotics, cocaine and stimulants; hallucinogen abuse; polydrugmania	Unemployment	On average, 11 years after the start of substance use, the majority of participants become unemployed.
USA, NESARC, 2001–2004/2012–2013, 23,446/23,346, ≥18	Okechukwu, C. et al., 2018 [38]	Past year marijuana use and use disorder, lifetime alcohol and drug disorder	Unemployment/ Employment	Past year marijuana use increases the risk of job loss by 50%, especially with daily use.
USA, FTDO, 1992–1994, n.a., n.a.	Chintakrindi, S. et al., 2015 [55]	Drug use prior to arrest (alcohol, marijuana, uppers, cocaine, crack, heroin, any drugs)	Unemployment/ Employment	Substance use is negatively associated with employment.
Finland, SF, 2000–2007, 204,422, 45–64	Paljärvi, T. et al., 2014 [42]	Alcohol-related death in relation to average annual number of days	Unemployment/ Employment	In total, 56% of alcohol-related deaths concern people who were employed ten years prior death; and this about two years less in comparison to people who died of other reasons and five years less in comparison to people who did not die.
USA, n.a. 1983/1985–1986/1992/1997/2002/2007/2012–2013, 528, ∅43	Brook, J. et al., 2014 [56]	Heavy/continuous smokers, late starters, occasional smokers, quitters/decreasers	Unemployment/ Employment	Continuous and occasional smoker tend to become unemployed in later years.
USA, CLS, 2004–2005, 620, 17–19	Arria, A. et al., 2013 [58]	Alcohol consumption, drug use (infrequent marijuana use, sporadic and persistent drug use)	Unemployment/ Employment	Young adults with drug use over persistent time might have higher risk of unemployment after college.
USA, HRS, 1992–2008, 20,557, ≥50	Deb, P. et al., 2012 [59]	Daily number of drinks	Unemployment/ Employment	People who show risky health patterns are particularly vulnerable for high rates of daily number of drinks in unemployment.
USA, n.a., n.a., 110, n.a.	Weber, E. et al., 2012 [44]	MA use characteristics (age of first use, total duration and quantity of use, last use, injection use) Non-MA dependence (cannabis, alcohol, cocaine, other substances)	Unemployment/ Employment	Neurocognitive deficits by methamphetamine use are associated with higher rates of unemployment.

DNHS: Danish National Health Survey, EDADES: Spanish Household Survey on Alcohol and Drugs in Spain, SUNRISE: Integrated Support of Unemployed at Risk of Substance Abuse Disorder, NESARC: National Epidemiologic Survey on Alcohol and Related Conditions, FTDO: First Time Drug Offender Program, SF: Statistics Finland, CLS: College Life Study, HRS: Health and Retirement Survey.

**Table 3:** Effects of Job Loss/Unemployment on Substance-Use Disorders.

Data Source, N, Age	Authors	Outcome Measures	Effects
U.K., MCS/ALSPAC/NS/BCS70/NCDS/USOC/ELSA/GS, 2020, 27,841, 16–66	Green, M. et al., 2022 [45]	Current smoking, vaping, and drinking among furloughed, no longer employed and stable unemployed people	Only correlation between stable unemployment women and smoking (ARR * = 1.35) as well as no longer employed women and vaping (ARR = 2.74).
Netherlands, DLCS, 2006–2013, 45,967, 25–50	Mangot-Sala, L. et al., 2021 [34]	Abstainers, moderate/heavy/binge drinking	Only correlation between long-term unemployment and heavy as well as binge drinking. Moreover, some short-term unemployed individuals are more likely to become abstinent.
USA, TEDS, 1993–2016, n.a., age ≥ 18	Azagba, S. et al., 2021 [48]	Treatment admissions for primary substance abuse (opiates, cocaine, alcohol, marijuana, other drugs, stimulants)	For every unit of rising unemployment rate, opiate treatment admissions increase by 9%. Other substances are associated with similar results; only stimulants show negative correlation.
Sweden, SWIP, 2005–2017, 2,743,764, 18–61	Jonsson, J. et al., 2021 [26]	Substance abuse	Unemployment is associated with higher risk of developing substance abuse (HR ** = 1.05–2.19).
England, GP, 2011–2017, 8736, n.a.	Vandoros, S. et al., 2020 [49]	Opioid prescribing	For every percentage point of rising unemployment rate, defined daily dose of opioid per capita increase by 0.017 (0.9% compared to the average).
USA, CARDIA, 2005–2010, 1307, n.a.	Swift, S. et al., 2020 [50]	Binge drinking, past 30-day drug use	Negative employment changes are associated with decreasing binge drinking, but increasing drug use in the past 30 days.
Italy, n.a., 2019, 314, n.a.	De Sio, S. et al., 2020 [41]	Alcohol-related biomarkers (carbohydrate-deficient transferrin, aspartate transaminase, alanine transaminase, γ-glutamyltransferase, mean corpuscular volume)	Office worker show lower rates in alcohol-related biomarkers then unemployed people.
USA, n.a., n.a., 553, ≥18	Miguel, A. et al., 2019 [43]	History of cocaine use and substance abuse treatment, concomitant psychiatric (alcohol-use) disorders	Stable unemployment during treatment of cocaine use is associated with negative outcomes. In contrast, being employed during treatment is associated with better outcomes.
Spain, EDADES, 2013, 14,113, 25–64	Teixidó-Compañó, E. et al., 2018 [32]	Hazardous drinking, heavy cannabis use, hypnosedative consumption	Unemployed people, no matter if male or female, show higher rates of cannabis and hypnosedative use, whereas unemployed women show less rates in alcohol drinking.
Netherlands, HIS, 2004–2013, 26,355, 30–64	De Goeij, M. et al., 2017 [53]	Episodic drinking before and during 2008 economic crisis	Job loss during economic crisis is only associated with chronic (OR 1.43/OR 1.42), but not with episodic drinking.
USA, PSID, 1999–2011, 41,231, n.a.	Grafova, I. et al., 2017 [31]	Current and former smoker, smoking relapse, increased and decreased cigarette consumption, quit smoking	Unemployment initially decreases the risk of cigarette consumption and its relapse in men, but not in long term.
Spain, Flash EB 401, 2011/2014, n.a., 15–24	Ayllón, S. et al., 2017 [29]	Consumption of cannabis and new substances	For 1% of rising unemployment rate, consumption of cannabis at any point in time among young people increases by 0.7%.
Germany, SUF, 2016, 8951, 15–64	Hollederer, A. et al., 2016 [27]	Consumption of beer, wine, alcoholic mixed drinks, spirits, and nicotine	Twice as high rate of unemployed smokers as employed smokers; but alcohol consumption rates are higher in employed people.
AT/BE/CZ/DK/FR/ DE/IT/NL/ES/SE/CH ***, SHARE, 2006–2012, 7615, 50–64	Bosque-Prous, M. et al., 2015 [39]	Hazardous drinking	Increasing unemployment rate leads to increasing rates of hazardous drinking by 32%.
USA, SSDP, n.a., n.a., 22–33	Lee, J. et al., 2015 [54]	Heavy episodic drinking, daily cigarette smoking, and marijuana use	Unemployment among young adults is associated with heavy episodic drinking. This does not apply to marijuana use and just possibly to daily cigarette smoking.
USA, NSDUH, 2002–2004/2005- 2007/2008/2009- 2010, ~405,000, ≥18	Compton, W. et al., 2014 [28]	Heavy alcohol, illicit drug and tobacco use; alcohol and illicit drug abuse or dependence	Unemployed people tend to higher rates in all mentioned outcome measures; also during economic crisis.
USA, NAS12, 2009–2010, 5382, ≥18	Mulia, N. et al., 2014 [33]	Alcohol-related health problems prior to recession	Job loss is associated with higher risk of alcohol-related health problems in men, especially drunkenness, its consequences and alcohol dependence.
USA, NESARC, 2001–2002/2004–2005, n.a., 19–60	Baldwin, M. et al., 2013 [57]	Alcohol and drug disorder	Unemployed people with alcohol disorder in wave I have a 3.7 respectively 8.8 percentage point higher chance of finding part-time respectively full-time work in wave II. Part-time employed people with alcohol disorder in wave I have a 6.1 percentage point higher chance of continuing in their work in wave II while those with drug disorder have a 13.8 percentage point lower chance of this work in wave II.
Germany, SOEP, 1998–2009, 52,940, 17–65	Schunck, R. et al., 2012 [30]	Smoking and number of cigarettes per day	Unemployment increases the risk of smoking, but not its intensity.
USA, NESARC, 2001–2002/2004–2005, 34,120, ≥18	Dávalos, M. et al., 2011	Alcohol consumption (binge drinking and its number of days, driving after too much to drink, abuse/dependence)	Unemployment is associated with rising alcoholic (binge) drinking events as well as its abuse/dependence.

MCS: Millennium Cohort Study, ALSPAC: Avon Longitudinal Study of Parents and Children (G0 = parents, G1 = children), NS: Next Steps, BCS70: 1970 British Cohort Study, NCDS: National Child Development Study, USOC: Understanding Society, ELSA: English Longitudinal Study of Aging, GS: Generation Scotland, DLCS: Dutch Lifelines Cohort Study, TEDS: Treatment Episode Data Set, SWIP: Swedish Work, Illness, and Labour-market Participation, GP: General Practice Data Dashbord, CARDIA: Coronary Artery Risk Development in Young Adults, EDADES: Spanish Household Survey on Alcohol and Drugs, HIS: Health Interview Survey, PSID: Panel Study of Income Dynamics, Flash EB 401: Flash Eurobarometer 401 “Young people and drugs”, Survey on Alcohol and Related Conditions, SUF: Scientific Use File, SHARE: Survey of Health, Ageing and Retirement in Europe, SSDP: Seattle Social Development Project, NSDUH: U.S. National Survey on Drug Use and Health, NAS12: U.S. National Alcohol Survey, NESARC: National Epidemiologic Survey on Alcohol and Related Conditions, SOEP: German Socio-Economic Panel, * ARR: Absolute Risk Reduction, ** HR: Hazard Ratio, *** AT/BE/CZ/DK/FR/DE/IT/NL/ES/SE/CH: Austria, Belgium, Czech Republic, Denmark, France, Germany, Italy, Netherlands, Spain, Sweden, Switzerland.

**Table 4:** Unemployment as a risk factor of relapses after treatment.

Data Source, N, Age	Authors	Outcome Measures	Effects
U.K., MCS/ALSPAC/NS/BCS70/NCDS/USOC/ELSA/GS, 2020, 27,841, 16–66	Green, M. et al., 2022 [45]	Smoking/vaping more–incl. relapse and initiation	In five out of seven surveys, unemployed participants tend to higher rates of smoking (incl. relapse and initiation) while in five out of six surveys, they tend to more vaping (incl. relapse and initiation).
USA, PSID, 1999–2011, 41,231, n.a.	Grafova, I. et al., 2017 [31]	Smoking relapse	In total, 10% of the unemployed participants who smoked in the past relapse until the second wave.
Germany, SOEP, 1998–2009, 52,940, 17–65	Schunck, R. et al., 2012 [30]	Smoking and number of cigarettes per day	Unemployed people do not seem to show changes in smoking relapse.

MCS: Millennium Cohort Study, ALSPAC: Avon Longitudinal Study of Parents and Children (G0 = parents, G1 = children), NS: Next Steps, BCS70: 1970 British Cohort Study, NCDS: National Child Development Study, USOC: Understanding Society, ELSA: English Longitudinal Study of Aging, GS: Generation Scotland, PSID: Panel Study of Income Dynamics, SOEP: German Socio-Economic Panel.

**Table 5:** Unemployment reducing the success of smoking cessation.

Data Source, N, Age	Authors	Outcome Measures	Effects
UK, MCS/ALSPAC/NS/BCS70/NCDS/USOC/ELSA/GS, 2020, 27,841, 16–66	Green, M. et al., 2022 [45]	Smoking/vaping less–incl. cessation	In two out of seven surveys, unemployed participants tend to lower rates of smoking (incl. cessation) while in one out of six surveys, they tend to less vaping (incl. cessation).
USA, PSID, 1999–2011, 41,231, n.a.	Grafova, I. et al., 2017 [31]	Quit smoking	A total of 1/5 of the unemployed participants who smoked in the past quit smoking until the second wave.
USA, n.a. 1983/1985–1986/ 1992/1997/2002/ 2007/2012–2013, 528, ∅43	Brook, J. et al., 2014 [56]	Smoking quitters	Regarding unemployment, there seem to be no differences between smoking quitters and those who do not smoke.
Germany, SOEP, 1998–2009, 52,940, 17–65	Schunck, R. et al., 2012 [30]	Smoking and number of cigarettes per day	Unemployed people do not seem to show changes in smoking cessation.

MCS: Millennium Cohort Study, ALSPAC: Avon Longitudinal Study of Parents and Children (G0 = parents, G1 = children), NS: Next Steps, BCS70: 1970 British Cohort Study, NCDS: National Child Development Study, USOC: Understanding Society, ELSA: English Longitudinal Study of Aging, GS: Generation Scotland, PSID: Panel Study of Income Dynamics, SOEP: German Socio-Economic Panel.

**Table 6:** Substance-use patterns based on unemployment rates and business cycle/cyclical fluctuations.

Data Source, N, Age	Authors	Dependent Variables	Effects
USA, TEDS, 1993–2016, n.a., ≥18	Azagba, S. et al., 2021 [48]	Treatment admissions for primary substance abuse (opiates, cocaine, alcohol, marijuana, other drugs, stimulants)	Correlation between unemployment rates and annual substance abuse admissions has the same causal direction during economic crisis and during times without crisis.
Spain, EDADES, 2007/2013, 23,258/22,862, 16–64	Casal, B. et al., 2020 [40]	Consumption of cannabis, cocaine, and both of them	Cannabis and cocaine use have an higher impact on unemployment especially in economic recessions.
Netherlands, HIS, 2004–2013, 26,355, 30–64	De Goeij, M. et al., 2017 [53]	Episodic drinking before and during 2008 economic crisis	Job loss during economic crisis is associated with chronic alcohol use but not with episodic drinking.

TEDS: Treatment Episode Data Set, EDADES: Spanish Household Survey on Alcohol and Drugs in Spain, HIS: Health Interview Survey.

## Data Availability

All data are provided in this manuscript.
